# Local treatment of metastases plus systemic chemotherapy on overall survival of patients with metastatic nasopharyngeal carcinoma

**DOI:** 10.1002/hed.26706

**Published:** 2021-05-03

**Authors:** Wenjun Liao, Jinlan He, Qiheng Gou, Baofeng Duan, Ping Ai, Lei Liu, Yanchu Li, Kexing Ren, Min Yao, Nianyong Chen

**Affiliations:** ^1^ Department of Head and Neck Oncology, Department of Radiation Oncology, Cancer Center and State Key Laboratory of Biotherapy, West China Hospital Sichuan University Chengdu Sichuan 610041 China; ^2^ Department of Radiation Oncology University Hospitals Cleveland Medical Center, Case Western Reserve University Cleveland Ohio USA

**Keywords:** chemotherapy, distant metastasis, local treatment, nasopharyngeal carcinoma, radiotherapy

## Abstract

**Background:**

To investigate the effect of local treatment of metastases on overall survival (OS) of patients with metastatic nasopharyngeal carcinoma (NPC).

**Methods:**

One hundred and forty‐seven patients were included. The association between local treatment and OS was examined with propensity score matching (PSM) method.

**Results:**

In entire cohort, the median OS was significantly longer in patients with local treatment of metastases plus chemotherapy compared to those with chemotherapy alone (71.7 vs. 16.2 months; *p* < 0.001). In PSM cohort, similar OS benefit of patients with local treatment was observed (55.6 vs. 17.6 months; *p* = 0.011). The survival benefit of local treatment remained regardless of the number of metastatic lesions and metastatic sites. Patients received radiation doses of >60 Gy had longer OS than those who received less.

**Conclusions:**

Local treatment of metastases could improve OS of patients with metastatic NPC and could be considered in their treatment in addition to chemotherapy.

## 1 INTRODUCTION

Nasopharyngeal carcinoma (NPC) is rare worldwide but endemic in southeast China.^1^ In recent years, with the use of intensity‐modulated radiotherapy (IMRT) and concurrent chemotherapy, locoregional control has been considerably improved, and distant metastasis has become the leading cause of mortality in patients with NPC.^2^ About 20%–30% of patients with locally advanced NPC develop distant metastasis after definitive chemoradiotherapy within 2–3 years.^3^, ^4^, ^5^

According to a phase III randomized clinical trial, gemcitabine plus cisplatin (GP) has been recommended as a standard treatment for these patients.^6^ However, the median progression‐free survival (PFS) was 7 months and only around 30% of patients were alive at 3 years.^6^ A few studies have indicated that incorporating local treatment of metastases such as local radiotherapy (RT) or surgery might bring a survival benefit to patients with distant metastasis compared to chemotherapy alone.^7^, ^8^, ^9^ Nevertheless, more robust evidence is needed to confirm these findings. Furthermore, critical issues such as optimal candidates for local treatment of metastases and appropriate radiation doses for metastases have not been explored adequately.

Therefore, the present study aims at determining the effect of local treatment of metastases plus systemic chemotherapy on overall survival (OS) of patients with metastatic NPC, further shedding light on the value of local treatment of metastases in these patients.

## 2 MATERIAL AND METHODS

### 2.1 Study population

**FIGURE 1:**
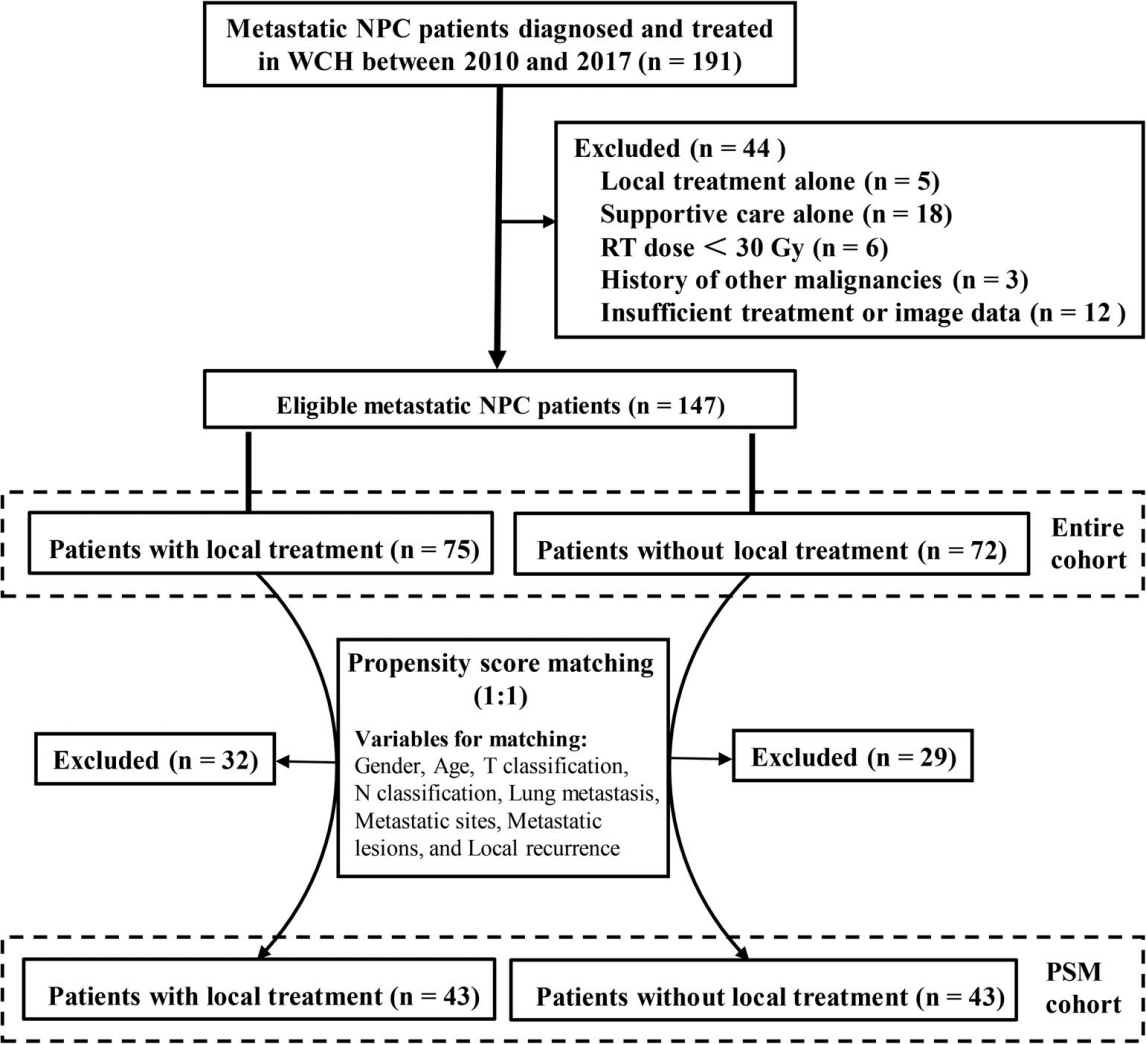
Flowchart for patient selection. NPC, nasopharyngeal carcinoma; PSM, propensity score matching; RT, radiotherapy; WCH, West China Hospital

Patients with NPC diagnosed at the West China Hospital (WCH) between January 2010 and December 2017 were retrospectively reviewed. Inclusion criteria of metastatic NPC were as follows: patients had pathologically confirmed NPC; patients were diagnosed as nonmetastatic NPC initially but were confirmed to have distant metastasis after definitive treatment; patients received systemic chemotherapy with or without local treatment of metastases. Exclusion criteria included patients with radiation doses of local treatment of metastases <30 Gy (doses were converted to equivalent dose in 2 Gy per fractionation [EQD_2_] assuming an *α*/*β* ratio of 10 for NPC), patients with insufficient treatment or image data, or patients with history of other malignancies. A total of 147 patients were identified and included in this study (Figure 1).

All patients in this study were restaged according to the 8th edition of the American Joint Committee on Cancer (AJCC). This study was approved by the Ethics Committee on Biomedical Research of the hospital and informed consent was waived.

### 2.2 Diagnosis and treatments

Metastatic status was confirmed via a biopsy of metastatic lesions or imaging tests including computed tomography (CT) for chest, ultrasonic/CT/MRI for abdomen, and whole‐body bone scan, or positron emission tomography/computed tomography (PET/CT) in selected patients. The initial treatment modalities of patients received before metastasis were listed in Table S1, Supporting Information.

All patients included in this study received chemotherapy. The median cycles of chemotherapy were 4 (range: 2–8 cycles). Cisplatin‐based regimens were used including cisplatin (80 mg/m^2^, Days 1–3) plus gemcitabine (1000 mg/m^2^, Days 1, 8) (GP), and cisplatin (80 mg/m^2^, Days 1–3) plus docetaxel (60–80 mg/m^2^, Day 1) or paclitaxel (135–175 mg/m^2^, Day 1) with or without 5‐flurouracil (600–1000 mg/m^2^, Days 1–5) (TP/TPF).

Of these 147 patients, 75 (51.0%) received local treatment of metastases in addition to chemotherapy. The administration of local treatment was mainly decided by the attending physicians under the premise that local treatment was technically feasible, with several factors including the number of metastasis, the location of metastasis, the sparing of organs at risk and the expected efficiency taken into consideration, to eliminate metastases or relieve symptoms. The techniques of local treatment of metastases were listed in Table 1. There were 64 (85.3%) patients received local treatment of metastases after chemotherapy, while 11 (14.7%) received local treatment before chemotherapy. For those with less than three metastatic lesions (*n* = 43), all metastatic lesions were treated with RT (*n* = 34) or surgery (*n* = 9). For those with more than three metastatic lesions (*n* = 32), local treatment was administrated only to the lesions that were resistant to systemic chemotherapy or threatened important functions of body.

**TABLE 1:** Techniques of local treatment of metastases in metastatic NPC patients in the entire cohort

Characteristic	No. of patients (%)
Treatment modalities	
Chemotherapy alone	72 (49.0)
Chemotherapy + local treatment	75 (51.0)
Sequence of local treatment and chemotherapy	
Chemotherapy first	64 (85.3)
Local treatment of metastasis first	11 (14.7)
Local treatment modalities of metastases	
Local radiotherapy alone	61 (81.3)
Surgery alone	9 (12.0)
Others^a^	5 (6.7)
Radiation technique of metastases (for those radiation alone)	
IMRT	58 (95.1)
3‐DCRT	3 (4.9)
Radiation fractionation of metastases (for those radiation alone)	
Conventional fractionation	38 (62.3)
Hypo‐fractionation^b^	23 (37.7)
Prescription for lung metastasis	
48 Gy/4 F	11 (44.0)
30–60 Gy/10–30 F	14 (56.0)
Prescription for bone metastasis	
30–50 Gy/10–25 F	10 (50.0)
>50–60 Gy/25–30 F	10 (50.0)
Prescription for liver metastasis	
48 Gy/4 F	5 (38.5)
30–60 Gy/10–30 F	8 (61.5)
Radiation doses of metastases (for those radiation alone)	
30–60 Gy^c^	27 (44.3)
>60 Gy^c^	34 (55.7)

Abbreviations: 3D‐CRT, three‐dimensional conformal radiation therapy; IMRT, intensive modulated radiation therapy; No., number; NPC, nasopharyngeal carcinoma.

^a^ Included radiotherapy plus vertebral plasty (*n* = 2), radiofrequency ablation (*n* = 2), and radiotherapy plus surgery (*n* = 1).

^b^ ≥3 Gy/fractionation.

^c^ Doses were converted to equivalent dose in 2 Gy per fraction (EQD_2_) assuming an *α*/*β* ratio of 10 for nasopharyngeal carcinoma.

In the 75 patients who received local treatment, 61 (81.3%) received RT alone. Of these, 58 (95.1%) were treated with intensive modulated radiation therapy (IMRT), and 23 (37.7%) received hypo‐fractionation RT with four to fifteen fractionations. The prescribed radiation doses administered to lung metastasis, bone metastasis, and liver metastasis were showed in Table 1. The radiation doses converted to EQD_2_ ranged from 30 to 95 Gy, with 34 patients receiving radiation doses of ≥60 Gy. Moreover, surgical resection of metastases was performed in five patients with lung metastasis, two with liver metastasis, and two with metastasis to parotid.

Twenty‐five patients presented with local recurrence in the entire cohort, and 7 of the 25 patients received treatment to local recurrence. The recurrence patterns and treatment modalities were showed in Table S2.

### 2.3 Follow‐up

Patients were evaluated every two cycles of chemotherapy, and followed up every 3 months in the first 2 years, thereafter, every 6 months until death or lost to follow‐up (the last follow‐up was December 31, 2019). Response evaluation was based on Response Evaluation Criteria in Solid Tumors 1.1 (RECIST 1.1). OS was defined as the interval from the time of metastasis confirmation to the time of death from any cause.

### 2.4 Statistics analysis

Statistics analysis was performed using SPSS 22.0 software package (IBM SPSS Inc., Armonk, NY). Pearson chi‐square test was conducted to compare categorical variables. Survival analysis was calculated by Kaplan–Meier method, and survival curves of different groups were compared by log‐rank test. Cox regression model with enter method was used to determine multiple prognostic factors associated with OS.

Propensity score matching (PSM) analysis was used to reduce selection bias (Figure 1). The selected covariates entering the propensity model included sex, age, T classification, N classification, lung metastasis, metastatic sites (specific metastatic locations including lung, liver, bone, etc.), metastatic lesions (metastatic tumors in other parts of the body except the primary), and local recurrence. In this study, one to one matching was performed using the nearest‐neighbor matching method, with a caliper of 0.02. A 2‐tailed *p‐*value <0.05 was considered as significant.

## 3 RESULTS

### 3.1 Characteristics of metastasis and propensity score matching

For all patients in the entire cohort, the median interval from completion of initial treatment to the detection of distant metastasis, defined as disease‐free interval (DFI), was 16 months (range: 4.1–97.1 months). The most commonly metastatic site was lungs, accounting for 51.7% (*n* = 76), followed by bones (*n* = 53, 36.1%), and liver (*n* = 45, 30.6%), and patients with single metastatic site (*n* = 95, 64.6%) and ≥two metastatic lesions in the metastatic sites (*n* = 111, 75.5%) were frequently seen (Table 2).

**TABLE 2:** Clinical characteristics of metastatic NPC patients and propensity score matching

Characteristics	Entire cohort (*n* = 147)			PSM cohort (*n* = 86)		
	CT alone (*n* = 72), No. of patients (%)	CT + local treatment (*n* = 75), No. of patients (%)	*p*‐value^a^	CT alone (*n* = 43), No. of patients (%)	CT + local treatment (*n* = 43), No. of patients (%)	*p*‐value^a^
Age, median (range)	46 (14–80)	46 (13–70)	0.379	46 (19–80)	46 (13–70)	0.829
Sex			0.600			0.476
Male	53 (73.6)	58 (77.3)		32 (74.4)	29 (67.4)	
Female	19 (26.4)	17 (22.7)		11 (25.6)	14 (32.6)	
ECOG performance status			0.742			0.510
0	25 (34.7)	28 (37.3)		16 (37.2)	19 (44.2)	
1	47 (65.3)	47 (62.7)		27 (62.8)	24 (55.8)	
T classification			0.555			0.149
T1‐2	18 (25.0)	22 (29.3)		9 (20.9)	15 (34.9)	
T3‐4	54 (75.0)	53 (70.7)		34 (79.1)	28 (65.1)	
N classification			0.949			0.802
N0‐1	16 (22.2)	17 (22.7)		11 (25.6)	10 (23.3)	
N2‐3	56 (77.8)	58 (77.3)		32 (74.4)	33 (76.7)	
Plasma EBV DNA (copies/ml)^b^						0.671
≤10^3^	23 (31.9)	22 (29.3)		15 (34.9)	13 (30.2)	
>10^3^	25 (34.8)	25 (33.4)		14 (32.6)	12 (27.9)	
Unknown^c^	24 (33.3)	28 (37.3)		14 (32.5)	18 (41.9)	
Bone metastasis						0.822
Yes	28 (38.9)	25 (33.3)		16 (37.2)	15 (34.9)	
No	44 (61.1)	50 (66.7)		27 (62.8)	28 (65.1)	
Lung metastasis			0.057			0.516
Yes	43 (59.7)	33 (44.0)		21 (48.8)	18 (41.9)	
No	29 (40.3)	42 (56.0)		22 (51.2)	25 (58.1)	
Liver metastasis			0.156			0.795
Yes	26 (36.1)	19 (25.3)		10 (23.3)	9 (20.9)	
No	46 (63.9)	56 (74.7)		33 (76.7)	34 (79.1)	
Distant nodal metastasis			0.518			1.000
Yes	15 (20.8)	19 (25.3)		10 (23.3)	10 (23.3)	
No	57 (79.2)	56 (74.7)		33 (76.7)	33 (76.7)	
No. of metastatic sites			0.024			0.323
Single	40 (55.6)	55 (73.3)		30 (69.8)	34 (79.1)	
Multiple	32 (44.4)	20 (26.7)		13 (30.2)	9 (20.9)	
No. of metastatic lesions			0.075			0.621
Single	13 (18.1)	23 (30.7)		10 (23.3)	12 (27.9)	
Multiple	59 (81.9)	52 (69.3)		33 (76.7)	31 (72.1)	
Chemotherapy cycles			0.224			0.272
≤3	30 (41.7)	24 (32.0)		20 (46.5)	15 (34.9)	
≥4	42 (58.3)	51 (68.0)		23 (53.5)	28 (65.1)	
DFI			0.218			0.131
≤16	39 (54.2)	33 (44.0)		25 (58.1)	18 (41.9)	
>16	33 (45.8)	42 (56.0)		18 (41.9)	25 (58.1)	
Local recurrence			0.011			0.501
Yes	18 (25.0)	7 (9.3)		4 (9.3)	6 (14.0)	
No	54 (75.0)	68 (90.7)		39 (90.7)	37 (86.0)	

Abbreviations: CT, chemotherapy; DFI, disease‐free interval (calculated from the completion time of initial treatment to the time of occurrence of distant metastasis); EBV, Epstein–Barr Virus; ECOG, Eastern Cooperative Oncology Group; No., number; NPC, nasopharyngeal carcinoma; PSM, propensity score matching.

^a^ *p‐*values were calculated by chi‐square test.

^b^ Plasma EBV DNA detected at the diagnosis of metastasis.

^c^ EBV DNA detection was refused by these patients.

The baseline characteristics of the two treatment groups were in good balance except the number of metastatic sites and local recurrence (Table 2). The proportion of patients with single metastatic site in the local treatment group was higher (73.3% vs. 55.6%; *p* = 0.024), while the proportion of patients with local recurrence was lower in the local treatment group (9.3% vs. 25.0%; *p* = 0.011). To reduce possible selection biases, a new cohort, the PSM cohort, was constructed using one to one matching (Figure 1). As a result, this new cohort included 43 pairs of patients with or without local treatment of metastases, thereby eliminating the differences of observed baseline variables (*p* > 0.05) (Table 2).

### 3.2 Survival analysis

**FIGURE 2:**
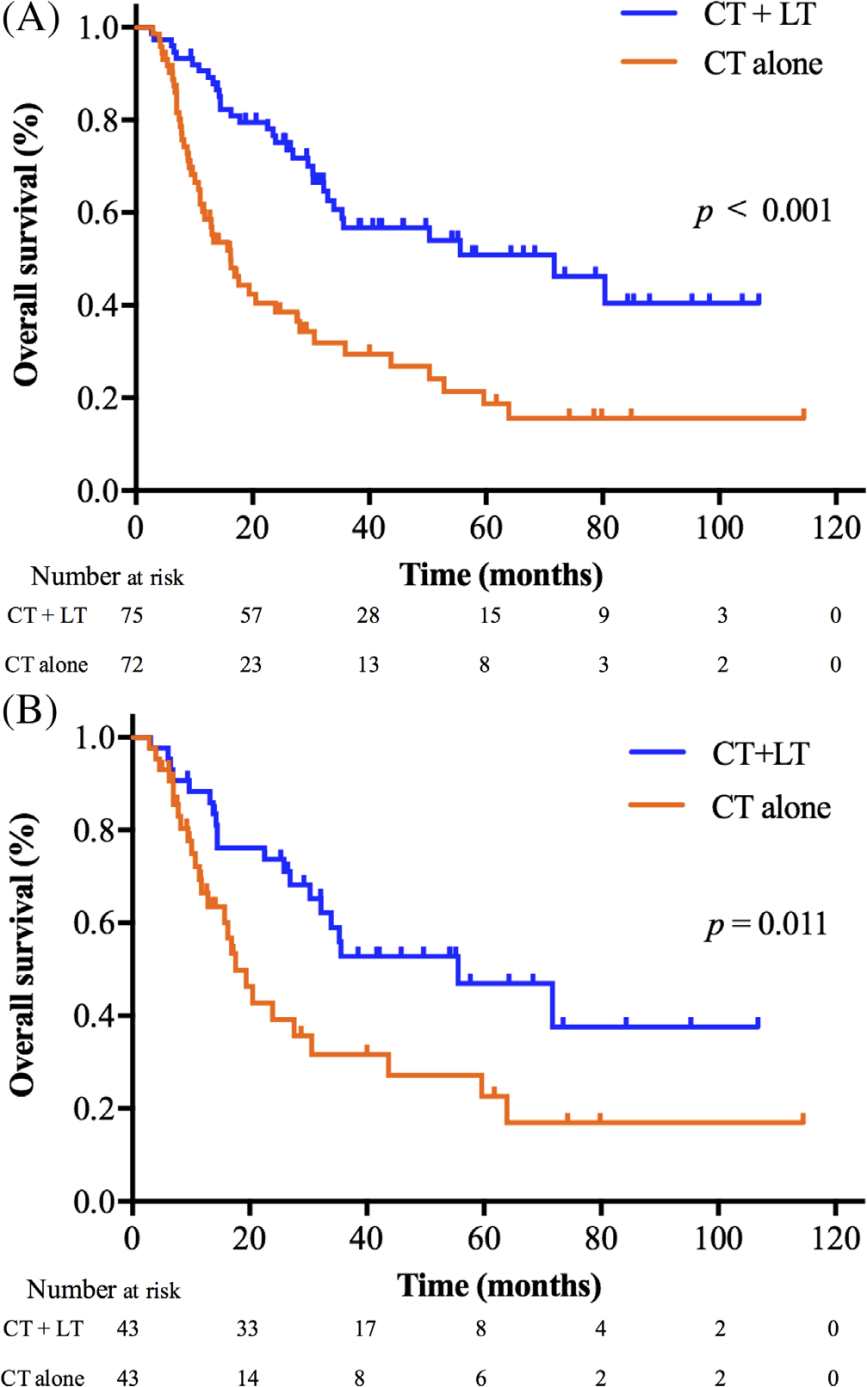
Overall survival curves stratified by treatment modalities in the entire cohort (A) and PSM cohort (B). CT + LT, chemotherapy and local treatment of metastases; CT, chemotherapy [Color figure can be viewed at wileyonlinelibrary.com]

For all patients in the entire cohort, the median follow‐up time from the detection of distant metastasis was 22.6 months (range: 2.7–114.5 months). Of the 147 patients, 33 (22.4%) had complete response, 45 (30.6%) had partial response, 29 (19.7%) had stable disease, and 40 (27.2%) had progressive disease. At last follow‐up, 80 (54.4%) patients had died. The 1‐, 3‐, and 5‐year OS of patients were 75.5%, 44.2%, and 35.5%, respectively. The median OS of patients with local treatment of metastases plus chemotherapy was significantly longer than that of patients with chemotherapy alone (71.7 vs. 16.2 months; 3‐year OS 55.4% vs. 25.9%; *p* < 0.001) in the entire cohort (Figure 2(A)). The benefit of local treatment of metastases on OS was further confirmed in the PSM cohort (55.6 vs. 17.6 months; 3‐year OS 50.6% vs. 32.5%; *p* = 0.011) (Figure 2(B)).

### 3.3 Univariate and multivariate analysis

A univariate analysis including all observed variables in the entire cohort was carried out. We found that bone metastasis, liver metastasis, number of metastatic sites, number of metastatic lesions, and local treatment of metastases were significantly associated with OS (*p* < 0.05) (Table 3). Therefore, a multivariate analysis including these variables was performed. Receiving local treatment of metastases was a positive independent factor for OS (HR 0.359, 95% CI 0.220–0.584; *p* < 0.001) (Table 3). Other independent prognostic factors that negatively influenced OS were bone metastasis (HR 2.374, 95% CI 1.387–4.063; *p* = 0.002), liver metastasis (HR 2.238, 95% CI 1.366–3.665; *p* = 0.001), and number of metastatic lesions (HR 3.228, 95% CI 1.482–7.029; *p* = 0.003). In the PSM cohort, consistent results were obtained (Table 4).

**TABLE 3:** Univariate and multivariate analysis of overall survival in the entire cohort

Characteristic	Univariate analysis			Multivariate analysis^a^		
	HR	95% CI	*p*‐value	HR	95% CI	*p*‐value
Sex (male vs. female)	1.149	0.697–1.894	0.586			
Age (>46 years vs. ≤46)	1.100	0.708–1.710	0.672			
T classification (T3‐4 vs. T1‐2)	1.093	0.673–1.774	0.720			
N classification (N2‐3 vs. N0‐1)	1.585	0.901–2.788	0.110			
Local recurrence (yes vs. no)	1.410	0.814–2.442	0.220			
DFI (≤16 vs. >16 months)	0.844	0.544–1.310	0.449			
Lung metastasis (yes vs. no)	0.797	0.513–1.238	0.313			
Bone metastasis (yes vs. no)	2.241	1.429–3.516	<0.001	2.374	1.387–4.063	0.002
Liver metastasis (yes vs. no)	2.139	1.372–3.336	0.001	2.238	1.366–3.665	0.001
Distant nodal metastasis (yes vs. no)	1.617	1.001–2.614	0.050	1.081	0.559–2.092	0.817
Metastatic sites (≥two vs. single)	2.462	1.579–3.841	<0.001	1.193	0.616–2.310	0.601
Metastatic lesions (≥two vs. single)	4.961	2.376–10.359	<0.001	3.228	1.482–7.029	0.003
Cycles of chemotherapy (>4 vs. ≤4)	0.847	0.536–1.338	0.476			
Local treatment of metastases (yes vs. no)	0.379	0.235–0.582	<0.001	0.359	0.220–0.584	<0.001

Abbreviations: CI, confidence interval; DFI, disease‐free interval (calculated from the completion time of initial treatment to the time of occurrence of distant metastasis); HR, hazard ratio.

^a^ Multivariate analysis included variables of bone metastasis, liver metastasis, distant nodal metastasis, metastatic sites, metastatic lesions, and local treatment of metastases.

**TABLE 4:** Univariate and multivariate analysis of overall survival in the PSM cohort

Characteristic	Univariate analysis			Multivariate analysis^a^		
	HR	95% CI	*p*‐value	HR	95% CI	*p*‐value
Sex (male vs. female)	0.962	0.504–1.835	0.906			
Age (years) (>46 vs. ≤46)	0.827	0.464–1.476	0.521			
T classification (T3‐4 vs. T1‐2)	0.762	0.411–1.413	0.388			
N classification (N2‐3 vs. N0‐1)	1.695	0.814–3.527	0.158			
Local recurrence (yes vs. no)	1.183	0.467–2.998	0.723			
DFI (≤16 vs. >16 months)	0.876	0.490–1.565	0.655			
Lung metastasis (yes vs. no)	0.483	0.262–0.890	0.020	0.780	0.356–1.706	0.533
Bone metastasis (yes vs. no)	2.393	1.333–4.295	0.003	2.200	0.873–5.546	0.095
Liver metastasis (yes vs. no)	1.809	0.963–3.398	0.065	3.114	1.352–7.171	0.008
Distant nodal metastasis (yes vs. no)	1.458	0.764–2.782	0.253			
Metastatic sites (≥two vs. single)	1.510	0.813–2.804	0.193	1.244	0.647–2.393	0.513
Metastatic lesions (≥two vs. single)	5.631	2.194–14.452	0.000	5.402	1.995–14.627	0.001
Cycles of chemotherapy (≤4 vs. >4)	0.780	0.429–1.418	0.415			
Local treatment of metastases (yes vs. no)	0.471	0.261–0.850	0.012	0.349	0.187–0.651	0.001

Abbreviations: CI, confidence interval; DFI, disease‐free interval (calculated from the completion time of initial treatment to the time of occurrence of distant metastasis); HR, hazard ratio; PSM, propensity score matching.

^a^ Multivariate analysis included variables of lung metastasis, bone metastasis, liver metastasis, metastatic lesions, and local treatment of metastases.

### 3.4 Factors that affect OS in patients received local treatment for metastases

**FIGURE 3:**
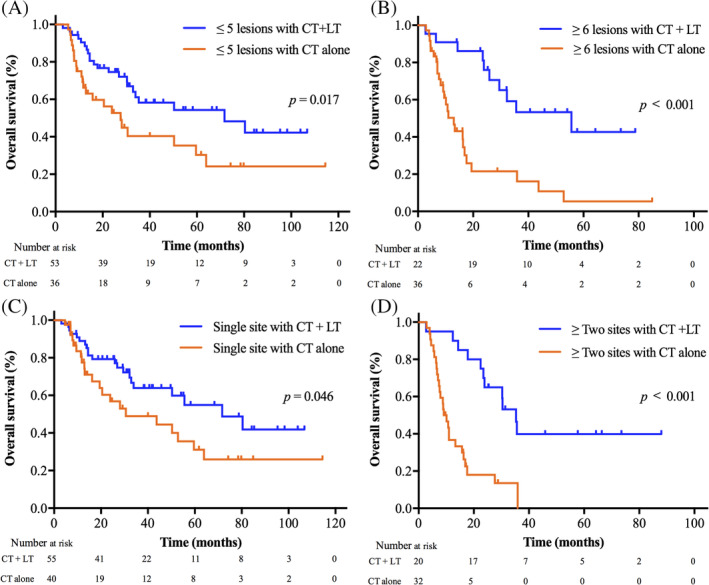
Overall survival curves stratified by treatment modalities in different number of metastatic lesions or metastatic sites: ≤five lesions (A) and ≥six lesions (B); single site (C) and ≥two sites (D). CT + LT, chemotherapy and local treatment of metastases; CT, chemotherapy [Color figure can be viewed at wileyonlinelibrary.com]

In order to identify which subgroups of patients with metastatic NPC benefited most from the local treatment of metastases, we further analyzed factors that might affect OS in those who had received local treatment in the entire cohort. Figure 3(A) showed that the median OS of patients with one to five metastatic lesions in the local treatment plus chemotherapy group was significantly higher than that of patients in the chemotherapy alone group (71.7 vs. 27.6 months; *p* = 0.017). Similarly, among patients with more than five lesions, patients who received local treatment plus chemotherapy also had longer median OS compared to those who received chemotherapy alone (55.6 vs. 13.0 months; *p* < 0.001) (Figure 3(B)). Moreover, for patients with single metastatic site, local treatment of metastases and chemotherapy extended the median OS of patients compared to chemotherapy alone (71.7 vs. 30.6 months; *p* = 0.046) (Figure 3(C)). Similarly, for patients with ≥two metastatic sites, the median OS of patients with local treatment of metastases was significantly higher than that of patients without it (35.3 vs. 9.6 months; *p* < 0.001) (Figure 3(D)).

**FIGURE 4:**
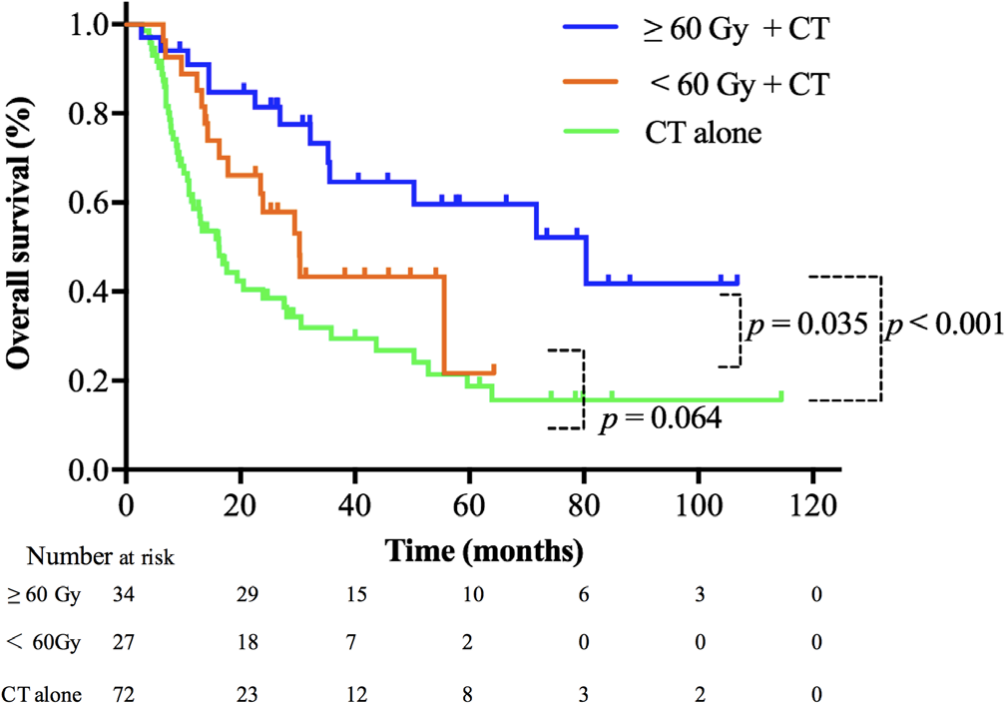
Overall survival curves stratified by treatment modalities and different radiation doses. Doses were converted to equivalent dose in 2 Gy per fractionation (EQD_2_) assuming an *α*/*β* ratio of 10 for nasopharyngeal carcinoma. CT, chemotherapy [Color figure can be viewed at wileyonlinelibrary.com]

Additionally, we analyzed the effect of radiation dose on the OS of patients with metastatic NPC using EQD_2_ 60 Gy as a stratification factor (Figure 4). When compared to patients who had chemotherapy alone, there was a trend of improved median OS in patients who received radiation doses of 30–60 Gy, although the difference was not statistically significant (30.3 vs. 16.2 months; *p* = 0.064). However, patients received 60 Gy or above showed a significant improved median OS compared to those received chemotherapy alone, with the median OS of 80.4 versus 16.2 months (*p* < 0.001). Furthermore, there is a significant improvement of median OS for patients received EQD_2_ ≥ 60 Gy as compared to those received EQD_2_ of 30–60 Gy (80.4 vs. 30.3 months, *p* = 0.035).

## 4 DISCUSSION

In this study, we examined the effect of local treatment of metastases on prognosis of patients with metastatic NPC. We demonstrated that the addition of local treatment of metastases to chemotherapy significantly improved OS of patients with metastatic NPC in the entire cohort, and our results of PSM cohort further confirmed the significant association between local treatment of metastases and an improved OS. Furthermore, multivariate analysis revealed that local treatment of metastases was an independent factor for predicting better OS of patients with metastatic NPC. The survival benefit remained even among patients with multiple metastases and those with ≥two metastatic sites. Moreover, we found that patients treated with an EQD_2_ of ≥60 Gy had longer OS compared to those with EQD_2_ < 60 Gy.

Systemic chemotherapy has been considered as an effective treatment for patients with metastatic NPC because of the relatively high objective response rate (ORR, 40%–65%).^10^, ^11^ Despite this, the outcomes of these patients are poor with a median OS of around 11–22 months when using platinum‐based chemotherapy as first‐line treatment.^6^, ^12^ Additionally, metastatic NPC is a heterogeneous disease with a wide range of survival.^13^ The metastatic site, the number of metastatic lesions, and their combinations will affect the survival of these patients.^14^ In some reports, a small proportion of patients with metastatic NPC had achieved long‐term survival.^15^, ^16^ However, the optimal treatment modalities for patients with metastatic NPC remain unclear.

Recently, there are published studies showing local treatment of metastatic lesions in addition to systemic chemotherapy improve OS of patients with metastatic NPC. One study used radiofrequency ablation (RFA) in patients with limited pulmonary metastases and showed that patients with RFA had a longer OS compared to those without (77.1 vs. 32.4 months; *p* = 0.009).^17^ Another study by Zheng et al. also showed that the 2‐year OS of patients with RT to metastases plus chemotherapy was longer than those with chemotherapy alone or best supportive care alone (57.7% vs. 32.7% vs. 1.6%; *p* < 0.01).^18^ Our study confirmed the survival benefit of local treatment of metastases in patients with metastatic NPC. We found that local treatment of metastases combined with chemotherapy significantly increased the median OS of patients with metastatic NPC by 38.0 months compared to those with chemotherapy alone in the PSM cohort, and reduced the risk of mortality by 34.9% in multivariate analysis. Comparable results were obtained in analysis of the entire cohort.

Oligometastasis, defined as those with one to five metastases,^19^ is considered as a transitional state between local invasion and extensive metastasis, where the metastatic ability of tumor is weak and the location and number of metastasis are limited.^20^ Mounting evidence demonstrated that local treatment, especially stereotactic body radiosurgery (SBRT) to metastases in these patients significantly improved survival in many solid tumors with low toxicity,^21^, ^22^, ^23^, ^24^ including oligometastatic prostate cancer^25^, ^26^ and non‐small cell lung cancer.^27^ Clinically, patients with oligometastasis or patients with one metastatic site are more prone to receive local treatment than those with multiple metastases. The role of local treatment in patients with multiple metastases is not clear since most of these patients are treated with chemotherapy alone. Therefore, we compared the OS of patients with metastatic NPC with or without local treatment using five metastases as a stratification factor. We found that local treatment plus chemotherapy resulted in an improvement of OS regardless the number of metastases or the metastatic sites. Even patients with more than five metastases or ≥two metastatic sites benefited from local treatment of metastases. It is possible that adding local treatment to those metastases that are insensitive to systemic chemotherapy or life threatening contributes to relieve symptoms, reduce tumor burden, elicit abscopal effect, and thus lead to a prolonged OS. Overall, our results confirmed that incorporation of local treatment of metastases was effective for achieving a longer survival in patients with metastatic NPC.

At present, the optimal radiation dose and fractionation scheme in the treatment of metastases remain largely unknown. One study revealed that patients with metastatic NPC received a BED (biologically effective dose) of ≥50 Gy had a more favorable outcome compared to those received less than 50 Gy.^18^ In our study, we found that although the median OS of patients was prolonged when receiving an EQD_2_ of 30–60 Gy plus chemotherapy compared to those treated with chemotherapy alone, the statistical difference was not significant. However, when they received EQD_2_ ≥ 60 Gy, the overall survival was significantly improved. And there was a statistically significant difference when compared those received EQD_2_ ≥ 60 Gy versus EQD_2_ of 30–60 Gy, indicating a higher radiation dose might be preferable. In our study, the median OS of patients received EQD_2_ ≥ 60 Gy more than doubled comparing to those received EQD_2_ of 30–60 Gy. Stereotactic body radiosurgery (SBRT) can offer highly conformal radiation to the targeted lesion with high dose per fraction and much shorter treatment course. SBRT should be a preferred radiation technique in local treatment of metastasis in future clinical application.

The sequence of local treatment of metastases and chemotherapy is also controversial. Some researchers have indicated that this had no significant influence on survival in patients with metastatic NPC. One study showed that for patients received local treatment of metastases before initiation of chemotherapy, the 3‐year OS was 45.0%, which was comparable to those who received initial chemotherapy followed by local treatment of metastases (3‐year OS, 50.2%).^8^ In our study, 85.3% of patients received chemotherapy before initiation of local treatment of metastases, which prevented further analyses on this issue. However, from the perspective of clinical practice, local treatment of metastases might ultimately depend on the number of metastatic lesions and metastatic sites and whether the metastatic lesions causing any symptoms. For oligometastasis, local treatment can be offered before initiation of chemotherapy. However, for patients with widespread metastases, local treatment can be directed to the lesions that are resistant to chemotherapy.

Our results showed that patients with lung metastasis had a more favorable outcome compared to those with bone metastasis or liver metastasis. Furthermore, patients with single metastatic lesion or single metastatic site had longer OS than those with multiple metastatic lesions or multiple metastatic sites, which were comparable with other studies.^14^, ^28^ These results further indicated that individualized treatment for patients with metastatic NPC is necessary. For patients with favorable clinical features, a curative intent involving systemic chemotherapy and ablative treatments to metastases is recommended.

Twenty‐five patients presented with local recurrence in the entire cohort, and 7 of the 25 patients received treatment to local recurrence. Previous studies have reported that radiotherapy to the primary could improve the OS of patients with NPC with metastasis at initial diagnosis,^29^, ^30^ which was consistent to our study showing that nasopharyngeal‐neck radiotherapy plus systemic chemotherapy markedly improved OS compared to systemic chemotherapy alone in this patient population.^31^ In patients who developed local recurrence alone post initial radical treatment, radiotherapy could also improve OS.^32^ For patients with both local recurrence and distant metastasis post treatment, like in our circumstance, little evidence has shown that local treatment to recurrence could improve the prognosis. Owing to the small number of patients with local recurrence and even smaller number of patients receiving local treatment to recurrence, it is quite difficult to analyze the effect of local treatment on prognosis. Fortunately, the similar proportion of patients between the two treatment groups after PSM and the minority of patients receiving local treatment to recurrence would help minimize the effect on OS.

This study has several limitations. Although patients with radiation doses of less than 30 Gy was excluded and PSM analysis was utilized, the selection bias was inevitable due to the retrospective nature of the study. Clinical characteristics of patients were well balanced statistically between groups after PSM, but in fact there were slightly less patients with local recurrence, multiple sites or multiple lesions in the local treatment group. The big difference (38 months) between the OS of the two groups somehow reflected this limitation, but our results were quite consistent with two other studies which revealed that local treatment of metastasis improved OS significantly by around 1 year^8^ and 3.7 years,^17^ respectively. These results would help provoke the interest in conducting randomized studies with selective homogeneous metastasis to clarify the role of local treatment to metastasis. In addition, due to the small sample size, issues on the sequence of local treatment of metastases and chemotherapy, and RT fractionation schemes were not fully addressed. Given these limitations, the results of the study need to be validated in a randomized clinical setting.

## 5 CONCLUSIONS

Taken together, we demonstrated that local treatment of metastases combined with chemotherapy improved OS of patients with metastatic NPC compared to chemotherapy alone. Therefore, we suggest that local treatment could be considered in the treatment of patients with metastatic NPC.

## AUTHOR CONTRIBUTIONS

*Analysis and interpretation of data, and drafting the article*: Wenjun Liao. *Analysis and interpretation of data, revising*: Jinlan He. *Analysis and interpretation of data*: Qiheng Gou. *Analysis and interpretation of data*: Baofeng Duan. *Analysis and interpretation of data*: Ping Ai. *Analysis and interpretation of data*: Lei Liu. *Analysis and interpretation of data*: Yanchu Li. *Analysis and interpretation of data*: Kexing Ren. *Revising*: Min Yao. *Conception and design*: Nianyong Chen. *Final approval of the version*: all authors.

## Supporting information


**Table S1** Initial chemotherapy regimens of patients received in definitive setting in the entire cohort.

**Table S2** Treatment modalities in patients with local recurrence.

## Data Availability

The data that support the findings of this study are available from the corresponding author upon reasonable request.
