# Supplementary material for: Local treatment of metastases plus systemic chemotherapy on overall survival of patients with metastatic nasopharyngeal carcinoma
Source: Head Neck. 2021 May 3;43(8):2423–33. doi: 10.1002/hed.26706 (PMC9539515; doi:10.1002/hed.26706)
Supplement: Supplementary file 1 — Table S1 Initial chemotherapy regimens of patients received in definitive setting in the entire cohort. [file HED-43-2423-s001.doc]

**Table S1** Initial chemotherapy regimens of patients received in definitive setting in the entire cohort.

| Characteristic | No. of patients (%) |
| --- | --- |
| Induction chemotherapy |  |
| Yes | 136 (92.5) |
| No | 11 (7.5) |
| Concurrent chemotherapy |  |
| Yes | 126 (85.7) |
| No | 21 (14.3) |
| Adjuvant chemotherapy |  |
| Yes | 99 (67.3) |
| No | 48 (32.7) |
| Induction chemotherapy regimens |  |
| Cisplatin+ taxane+ 5-FU (TPF) | 101 (74.3) |
| Cisplatin+ taxane (TP) | 17 (12.5) |
| Cisplatin+ gemcitabine (GP) | 15 (11.0) |
| Cisplatin+ 5-FU (PF) | 3 (2.2) |

Abbreviations: No., number.
