# Supplementary material for: Local treatment of metastases plus systemic chemotherapy on overall survival of patients with metastatic nasopharyngeal carcinoma
Source: Head Neck. 2021 May 3;43(8):2423–33. doi: 10.1002/hed.26706 (PMC9539515; doi:10.1002/hed.26706)
Supplement: Supplementary file 2 — Table S2 Treatment modalities in patients with local recurrence. [file HED-43-2423-s002.doc]

**Table S2** Treatment modalities in patients with local recurrence.

| Characteristic | No. of patients (%) |
| --- | --- |
| Site of local recurrence |  |
| Nasopharynx | 9 (36.0) |
| Cervical lymph node | 14 (56.0) |
| Nasopharynx and cervical lymph node | 2 (8.0) |
| Treatment of local recurrence |  |
| No | 18 (72.0) |
| Yes | 7 (28.0) |
| Treatment modalities for local recurrence |  |
| Radiotherapy* | 4 (57.1) |
| Surgery**^†^** | 3 (42.9) |

* 54 Gy/30 F to nasopharynx in 1 patient and 60 Gy/ 30 F to cervical lymph node in 3 patients.

**^†^** lymph node dissection for 3 patients with cervical lymph node recurrence.
